# Chronometric Administration of Cyclophosphamide and a Double-Stranded DNA-Mix at Interstrand Crosslinks Repair Timing, Called “Karanahan” Therapy, Is Highly Efficient in a Weakly Immunogenic Lewis Carcinoma Model

**DOI:** 10.3389/pore.2022.1610180

**Published:** 2022-05-27

**Authors:** Vera Ruzanova, Anastasia Proskurina, Yaroslav Efremov, Svetlana Kirikovich, Genrikh Ritter, Evgenii Levites, Evgenia Dolgova, Ekaterina Potter, Oksana Babaeva, Sergey Sidorov, Oleg Taranov, Alexandr Ostanin, Elena Chernykh, Sergey Bogachev

**Affiliations:** ^1^ Laboratory of Induced Cellular Processes, Institute of Cytology and Genetics of the Siberian Branch of the Russian Academy of Sciences, Novosibirsk, Russia; ^2^ Department of Natural Sciences, Novosibirsk National Research State University, Novosibirsk, Russia; ^3^ Common Use Center for Microscopic Analysis of Biological Objects SB RAS, Institute of Cytology and Genetics of the Siberian Branch of the Russian Academy of Sciences, Novosibirsk, Russia; ^4^ Oncology Department, Municipal Hospital No. 1, Novosibirsk, Russia; ^5^ Laboratory of Microscopic Research, State Research Center of Virology and Biotechnology “Vector”, Koltsovo, Russia; ^6^ Laboratory of Cellular Immunotherapy, Research Institute of Fundamental and Clinical Immunology, Novosibirsk, Russia

**Keywords:** cancer stem cells, Karanahan approach, Lewis lung carcinoma, committed tumor cells, tumor growth site, cytostatic agent, dsDNA, interstrand crosslink repair

## Abstract

**Background and Aims:** A new technology based on the chronometric administration of cyclophosphamide and complex composite double-stranded DNA-based compound, which is scheduled in strict dependence on interstrand crosslinks repair timing, and named “Karanahan”, has been developed. Being applied, this technology results in the eradication of tumor-initiating stem cells and full-scale apoptosis of committed tumor cells. In the present study, the efficacy of this novel approach has been estimated in the model of Lewis carcinoma.

**Methods:** To determine the basic indicative parameters for the approach, the duration of DNA repair in tumor cells, as well as their distribution along the cell cycle, have been assessed. Injections were done into one or both tumors in femoral region of the engrafted mice in accordance with the developed regimen. Four series of experiments were carried out at different periods of time. The content of poorly differentiated CD34^+^/TAMRA+ cells in the bone marrow and peripheral blood has been determined. Immunostaining followed by the flow cytometry was used to analyze the subpopulations of immune cells.

**Results:** The high antitumor efficacy of the new technology against the developed experimental Lewis carcinoma was shown. It was found that the therapy efficacy depended on the number of tumor growth sites, seasonal and annual peculiarities. In some experiments, a long-term remission has been reached in 70% of animals with a single tumor and in 60% with two tumors. In mice with two developed grafts, mobilization capabilities of both poorly differentiated hematopoietic cells of the host and tumor stem-like cells decrease significantly. Being applied, this new technology was shown to activate a specific immune response. There is an increase in the number of NK cell populations in the blood, tumor, and spleen, killer T cells and T helper cells in the tumor and spleen, CD11b+Ly-6C+ and CD11b+Ly-6G+ cells in the tumor. A population of mature dendritic cells is found in the tumor.

**Conclusion:** The performed experiments indicate the efficacy of the Karanahan approach against incurable Lewis carcinoma. Thus, the discussed therapy is a new approach for treating experimental neoplasms, which has a potential as a personalized anti-tumor therapeutic approach in humans.

## Introduction

Functionality of the human organism completely depends on different temporal cycles (circadian, seasonal, annual, etc.). At the cellular and molecular levels, this is manifested as time-dependent changes in the interplay of cellular life-supporting systems. Being a part of the organism, tumors also obey the biological timer, and processes occurring in tumor cells are affected accordingly [1]. It becomes increasingly clear that temporal patterns of molecular events and processes throughout the organism, organs, tissues and cells, including tumor ones, are critical for the successful treatment of a wide range of malignant neoplasms [2].

One of the effective approaches in therapy of different neoplasms is an immunotherapy. The latency and strength of the anti-tumor immune response depend on the complex of environmental factors (including temporal cycles), the status of the host and biological peculiarities of the tumor itself. In this regard, the paradigm, considering the individual immune status of the host organism as a starting point for subsequent immunotherapeutic interventions, has been stated [3].

These new concepts formed the basis for technologies related to the chronometric administration of therapeutic compounds with regard to the features of the tumor, immune cells and the general immune status of the host. The most prominent of these are *in situ* vaccination [4] and chronometric/metronomic chemotherapy with low doses of cyclophosphamide [5–8]. The main feature of the antitumor activity of these approaches is their independence from a specific target or molecule and, as a result, capability of both inducing an integral antitumor immune response and disrupting the protective properties of tumor-associated stroma.

Our group has developed the concept of “Karanahan technology.” This approach, similarly to those mentioned above, is of chronometric nature and implies the administration of both cyclophosphamide (CP) and the complex composite “DNAmix” preparation in strict dependence on the duration of DNA repair and the cell cycle pattern of each certain tumor. After being applied, the technology results in the eradication of cancer stem cells (CSCs) and the induction of the large-scale apoptosis of committed tumor cells.

### Brief History and Main Stages of the “Karanahan” Development

The history of this technology has began in 2006 during experiments with murine Krebs-2 carcinoma, and is associated with the following stages.

CSCs were found to be capable of native internalizing the fragments of double-stranded DNA (dsDNA). Carboxytetramethylrhodamine (TAMRA)-labeled polymerase chain reaction product of human AluI repeat has been designed and is still routinely used as a probe to detect DNA-internalizing cells. This feature allowed both the detection of stem-like cells in each certain tumor and tracing the changes in their content after treatments applied [9–18]. The possible mechanism of internalization is described in three our forthcoming reports, and is hypothesized to be associated with the interaction of dsDNA with heparin-binding domains in glycocalyx components specific for CSCs [19–26].

During the first experiments, it has been demonstrated that the process of repairing interstrand crosslinks (ICLs), caused by the effect of CP on Krebs-2 cells, has a 12 h-long time span of the escalation of double-strand breaks (DSBs) (nucleotide excision repair phase—NER), a 12 h-long plateau and a 12 h-long phase of homologous recombination (HR), during which the formed DSBs disappear.

At the first stage, ascites-bearing mice exposed to a single dose of CP received 12 hourly injections (one injection per hour) of dsDNA with subsequent intramuscular re-transplantation of ascitic cells into healthy mice. It was found that injections of native human dsDNA during the first 12 h after exposure to CP (the time of NER phase in Krebs-2 cells) abrogated the development of re-transplanted grafts. Injections of the same dsDNA during the HR phase (24–36 h after exposure to CP) had no effect and, as a result, re-transplanted grafts grew up normally. On the other hand, injections of chlormethine (nitrogen mustard)-treated salmon sperm dsDNA had an absolutely opposite effect: being injected during the HR phase, this compound abrogated the growth of grafts, while during the NER phase ascitic cell were tolerant to it. During these experiments, a lethal toxicity of both types of dsDNA has been determined. It was, however, found that toxicity of modified salmon sperm DNA could be reduced significantly by the dilution with native salmon sperm DNA, and such a dilution retained the capabilities of the preparation to prevent the development of re-transplanted grafts [15, 27, 28].

These experiments revealed that grafts do not grow if grafted ascitic cells are depleted of TAMRA+ CSCs due to the treatment applied [15].

Monotherapy with various DNA preparations (ssDNA, modified CCLssDNA, human DNA) or CP alone either had no therapeutic effect at all or it was negligible; the experimental animals have never been completely cured (the experimental data were reported sporadically in the references mentioned below.) Multiple combinations and variants of CP and dsDNA administration were tested. It was found that the process of repairing ICLs caused by the exposure of cells to CP can be arrested by two additional exposures, performed sequentially 1 hour before the end of each following repair round (36 h), and such a treatment results in complete asctites resorption. This treatment, however, resulted in the death of animals due to severe systemic inflammation, while in surviving mice, the tumor always recurred.

To resolve the issue of high toxicity of multiple dsDNA administration, we have proposed the following solution. We presumed that if we combine human dsDNA, salmon sperm dsDNA and modified salmon sperm dsDNA into a single preparation, and administer this preparation once at the time point delimiting two phases of ICLs repair, NER and HR (18 h after exposure to CP), then the following events should occur: 1) native human dsDNA would prevent the completion of the NER phase in one fraction of tumor cells, and 2) the mix of salmon sperm DNA would interfere with the HR phase in cells, which somehow have succeeded in completing the NER one. Already the first experiments with ascites-bearing mice, which were subjected to triple administration of CP and dsDNAmix, gave a complete ascites reduction and almost complete survival of animals without the development of systemic inflammation. In other words, we have found a non-toxic regimen of triple sequential administration of CP and DNAmix strictly associated with the DNA repair rounds.

Nevertheless, despite the visually complete reduction of ascites, all treated mice relapsed 14, 15 days after the therapy start. Tracing the events occurring with treated cells during 9 days revealed the following: 1) the treatment induces a large-scale apoptosis of committed tumor cells; 2) on a certain day, residual tumor cells accumulate in G2/M phase of the cell cycle. The percentage of TAMRA+ CSCs turned out to increase drastically at this time point. We have hypothesized that this expanded fraction of TAMRA+ CSCs is the subpopulation of Krebs-2 stem-like cells survived the treatment. An increased percentage of this type of cells over the background of a drastic decrease in the number of committed tumor cells (the bulk of tumor cells) testified for exactly the such events flow. This finding denoted the most important time point in the therapy, when all remaining tumor cells, both committed and stem-like, are synchronized in G2/M phases [29–33].

CP-induced ICLs, formed while cells are in these phases, can be “detected” by the cell only after entering a new G1 phase after mitosis [34]. At the same time, we have presumed (and later confirmed experimentally) that cells in G2/M phases are incapable of internalizing dsDNA due to cytoskeletal remodeling [15, 35]. Thus, it was suggested to add the fourth administration of CP at this “synchronization” time point, followed by the fourth administration of DNAmix during the upcoming G1 that would ensure wiping out the residual CSCs and committed tumor cells.

Due to the treatment applied, 75% of mice with ascitic and solid forms of Krebs-2 were cured completely. ∼25% of the mice died due to severe systemic inflammation and ∼50% of the animals survived with no relapse until the end of the monitoring span (180 days from the experiment beginning). Moreover, two females born healthy offspring [33,36–40]. Over 20 years of research, countless experiments with all possibles controls in every case have been conducted.

**FIGURE 1:**
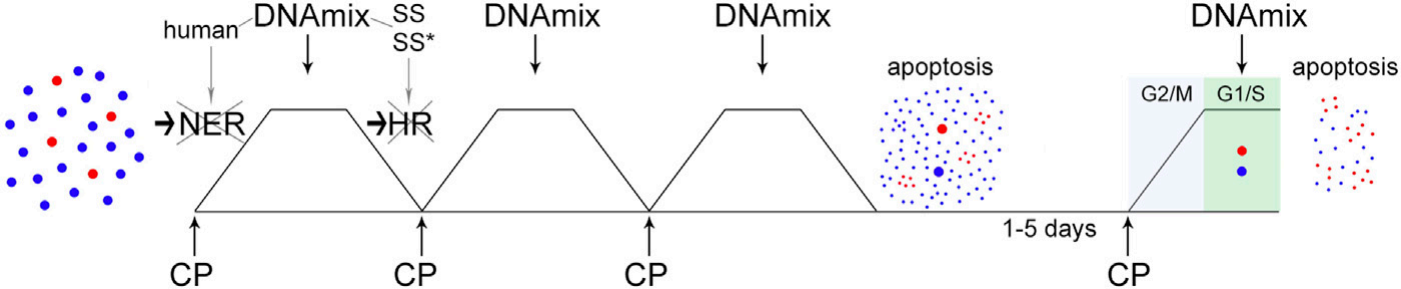
The concept scheme of “Karanahan”. At the first stage, tumor cells are subjected to triple exposure to CP and DNAmix, which are administered in strict dependence on ICLs repair timing. As a result of this procedure, the majority of tumor stem-like cells are eliminated over the background of large-scale apoptosis of the committed ones. At the same time, residual tumor stem-like and committed cells synchronize in the G2/M phase of the cell cycle. At the second stage, the fourth administration of CP and DNAmix (also linked to the repair process) is performed at the moment of synchronous entry of these cells into the G1 phase. As a result, all residual tumor cells, both stem-like and committed, are eliminated completely. A more detailed description of the molecular events accompanying this process is provided in the body of the manuscript. In the figure, the blue dots schematically indicate the committed tumor cells, the red ones are the stem tumor cells.

As a result, a technological approach named “Karanahan” (from the Sanskrit kāraṇa [“source”] + han [“to kill”] 
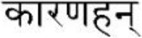
) has been developed. The general schematics of the technology with all essential elements is shown in Figure 1.

The current study reports the results of using a new approach to cancer treatment in curing mice with Lewis carcinoma. The results obtained indicate the high effectiveness of the therapy, which allows curing experimental animals from fulminant incurable carcinoma. The main outcome of the study is that, along with eradication of CSCs and tumor mass reduction, this therapeutic intervention activates the anti-tumor immunity and negates the immunosuppressive effect of tumor-associated stroma.

## Materials and Methods

### Experimental Animals

We used male and female 2- to 6-month-old C57BL/6 mice (weight, 18–24 g) bred at the Common Use Center Vivarium for Conventional Animals of the Institute of Cytology and Genetics of the Siberian Branch of the Russian Academy of Science (Novosibirsk, Russian Federation). Animals were kept in groups of 6–10 mice per cage with free access to food and water. All experiments with animals were conducted in strict compliance with the principles of humanity in accordance with the European Community Council Directives (86/609/EEC) and were approved by the Animal Care and Use Committee of the Institute of Cytology and Genetics SB RAS (protocol N 8 from 19 March 2019). Tumor burden did not exceed the recommended dimensions (maximum allowable size was 12, 13 mm in diameter); animals were sacrificed using the method of cervical dislocation.

### Tumor Model

Lewis carcinoma is a weakly immunogenic tumor characterized by high metastatic activity [41]. Intramuscular engraftment of 2 × 10^6^ cells is performed into femoral region of C57BL/6 mice. Carcinoma metastasizes to the lungs in 100% of cases. Lewis lung carcinoma occurred spontaneously in C57BL/6 mice in 1951. The strain was received from the National Cancer Institute of the United States in September 1973. The tumor consists of polymorphic cells, most of which are round. A representative image of the Lewis carcinoma cell culture is provided in Supplementary Figure S3. The standard culture medium for Lewis сarcinoma cells is DMEM/F-12 (1:1) medium (Biolot, Saint Petersburg, Russia) supplemented with 20% fetal bovine serum (Capricorn Scientific, Ebsdorfergrund, Deutschland), 100 μg/ml gentamicin (Dalkhimpharm, Khabarovsk, Russia) and 1 μg/ml amphotericin B (Synthesis, Kurgan, Russia).

### Counting TAMRA+ Cells in the Tumor in Mouse Blood and Bone Marrow

All procedures of TAMRA–DNA probe preparation and incubation with cells were performed as described in [15]. Cell content was analyzed using either microscopy or flow cytometry. Microscopy was conducted using a Zeiss Axio Imager M2 (Carl Zeiss Microscopy, Oberkochen, Deutschland). Flow cytometry was carried out using a BD FACSAria™ III Cell Sorter (BD, Franklin Lakes, United States).

### Assessment of the Content of TAMRA+ CD34^+^ Cells in the Population of Blood Mononuclear Cells

Mice were sacrificed by cervical dislocation and further decapitated to collect blood. The collected blood was immediately added with EDTA up to the final concentration of 7 mM. Erythrocytes were lysed with 130 mM ammonium chloride followed by the isolation of a mononuclear fraction. A total of 5 × 10^5^ freshly isolated mononuclear cells were incubated with 0.1 μg of Alu–TAMRA DNA for 10 min. Then, 2 μg of FITC rat anti-mouse CD34 (BD, Franklin Lakes, United States), FITC Rat IgG2a κ isotype control (BD, Franklin Lakes, United States) were added; the mixture was incubated for 1 h in the dark at room temperature. Cells were pelleted at 400 g and 4°C for 5 min and washed with PBS (Medigen, Novosibirsk, Russia); flow cytometry was performed on a BD FACSAria™ III Cell Sorter.

### Assessing the Length of DNA Repair Cycle in Tumor Cells

Tumor cells were incubated with 1 μg/ml mitomycin C (Sigma-Aldrich, St. Louis, United States) for 1 h at 37°C in a CO_2_ incubator (Memmert GmbH + Co. KG, Schwabach, Germany). The medium was then changed to the standard one, and samples were taken for analysis every 6 h. Repair cycle was estimated using the comet assay [42, 43] as described in [15]. Comet tail length (TM—tail moment) was evaluated using the CASP software (CASP, Wrocław, Poland).

### Assessing Cell Cycle After Triple Exposure to the Crosslinking Agent

Mice bearing tumors of ∼500–1000 mm^3^ were injected with 100 mg/kg of cyclophosphamide (Baxter, Halle, Germany) intraperitoneally three times with a 28-h interval. The identity of the ICL formation time for MMC *in vitro* and CP *in vivo* had been experimentally demonstrated earlier [37]. On the third day after the onset of cyclophosphamide injections, Lewis carcinoma cells were isolated from three mice to *in vitro* culture. On days 3–7 after the first exposure to the crosslinking agent, tumor cells were sampled for cell cycle profiling. The procedure was carried out using the standard propidium iodide (Thermo Fisher Scientific, Waltham, United States) protocol as described in [33] on the BD FACSAria™ III Cell Sorter and the BD FACSDiva™ Software (BD, Franklin Lakes, United States).

### Analysis of Changes in the Percentage of TAMRA+ Cells After Triple Exposure to the Crosslinking Agent

Lewis carcinoma cells were isolated from mice and incubated in standard medium at 37°C in a CO_2_ incubator overnight. The percentage of TAMRA+ cells was determined at the initial time point and on days 4–7 in the control sample and after triple treatment with 1 μg/ml mitomycin C in a serum-depleted medium for 1 h with a 28-h interval.

### Composite dsDNA

The composite dsDNA preparation (DNAmix) is a mixture of native and modified genomic DNA isolated from human placenta and salmon sperm and fragmented to a size of 200–6000 b.p. DNAmix is the subject of industrial property of KARANAHAN LLC [44].

### Treatment of Lewis Carcinoma

**FIGURE 2:**
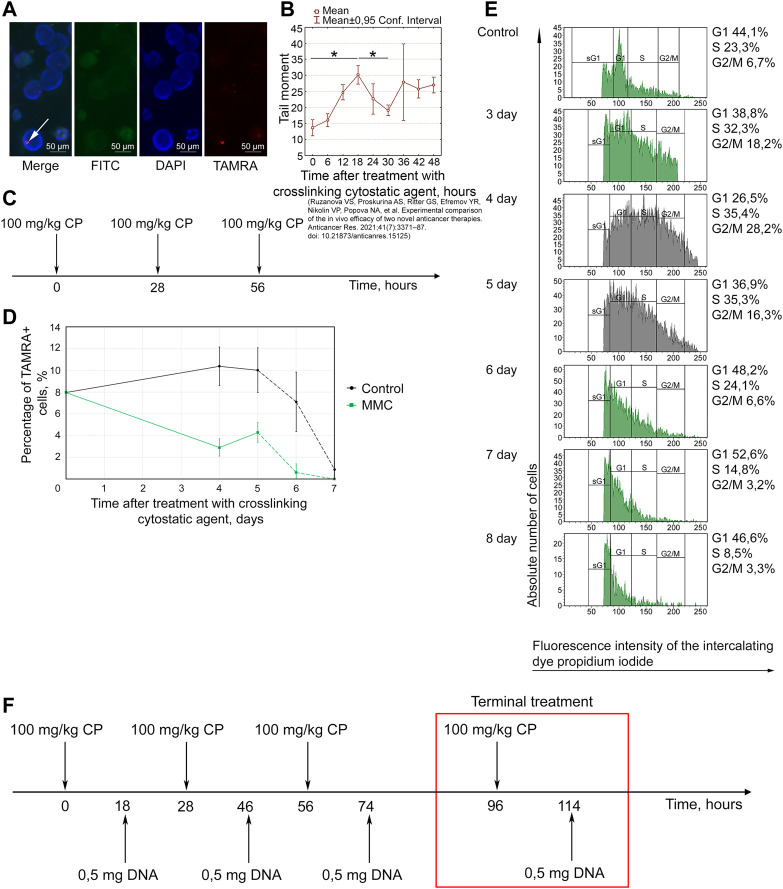
Basic parameters of the Karanahan approach for treating Lewis carcinoma. **(A)** TAMRA+ cells. The graphs show FITC, DAPI, and TAMRA channels and the merged image (Merge). Arrows point to the TAMRA-specific signal. Images were taken at ×15 magnification. Scale bars = 50 µm. **(B)** Analysis of the cell repair cycle. Changes in the number of double-strand breaks in tumor cells after incubation with mitomycin C assessed using the comet assay [38]. *—differences between points are significant with a probability of *p* < 0.001, Mann–Whitney U test. **(C)** Therapeutic regimen developed based on repair cycle parameters. **(D)** Changes in TAMRA+ cells in control and after triple treatment with mitomycin C. A decrease in the content of TAMRA+ cells was observed both in the experimental samples and control on day 6, which was apparently associated with non-viability of Lewis carcinoma cells in culture. The curves representing percentage of TAMRA+ cells after 5 days are shown by the dotted line. **(E)** Cell distribution by cell cycle phases after triple treatment of mice with cyclophosphamide. Control—Lewis carcinoma cells before treatment. The Y-axis shows the absolute number of cells. **(F)** Schematic representation of therapy in C57BL/6 mice grafted with Lewis carcinoma, including injections of 100 mg/kg of cyclophosphamide and 0.5 mg of DNAmix.

The experiments were carried out in Novosibirsk (55°02′29″ north latitude; 82°56′04″ east longitude). A total of 2 × 10^6^ Lewis carcinoma cells were grafted intramuscularly in either right femoral region or both femoral regions of mice. When the tumor reached 64–343 mm^3^ in size, mice were injected with the agents. The experimental group received 100 mg/kg of cyclophosphamide intraperitoneally and 0.5 mg/mouse of DNAmix intratumorally only in the right tumor, in accordance with the selected therapeutic regimen for Lewis carcinoma (Figure 2F). The group of tumor-bearing mice received similar injections of saline. During the experiment, tumor size, time of relapse occurrence, and mouse life expectancy were measured. Tumor sizes were measured using a caliper, and the tumor volume was calculated as the product of three measurements (h × l × w). At the end of one of the experiments, lungs were isolated from mice and fixed in 4% paraformaldehyde. After fixation, lung metastases were counted using a binocular microscope.

Specific sets of antibodies were used for immunophenotyping of the cellular composition of tumor-associated stroma, spleen, mononuclear fraction of peripheral blood, and peritoneal macrophages on days 15, 17 and 22 since the experiment beginning. Treated animals with residual detectable tumors were examined on days 15 and 22, animals with completely reduced tumors were examined on day 17. The untreated tumor-bearing animals in the control group that had survived were examined at each control point. On day 31, the activity of peritoneal macrophages was assayed. The main challenge was to determine the time point and cellular targets of responsive immune cells, which would be suitable for the analysis proposed. Ten specific molecular markers were used for the simultaneous analysis of five specific cellular subpopulations. Due to technical difficulties, only one animal from each group at every time point was used for immunophenotyping, which was sufficient for preliminary conclusion.

The following parameters were compared during the analysis: 1) cellular subpopulations between treated and untreated animals; 2) time-dependent changes within the individual subpopulations of cells, both within each group and between different ones; 3) the representation of certain subpopulations in different anatomical structures (tumor vs. spleen, tumor vs. peritoneal fraction, tumor vs. mononuclear fraction of peripheral blood), both within each group and between different ones; 4) cytolytic activity of peritoneal macrophages towards Lewis carcinoma cells.

To establish the statistical reliability, experiments were repeated using three animals from each group. In these experiments, animals were sacrificed on an appropriate day, tissues and organs were isolated and cellular subpopulation were analyzed as described above.

### Analysis of Changes in the Number of Immune Cell Populations in the Tumor, Spleen, and Among Blood Mononuclear Cells and Peritoneal Macrophages

To assess the immune response after therapy, the tumor, spleen, blood mononuclear cells, and peritoneal macrophages were isolated on days 15, 17 (except for tumor material, since tumor in the femoral area was completely resorbed in the mouse examined on day 17) and 22 after treatment initiation. To analyze populations of T regulatory lymphocytes and killer T cells, cells were fixed in an equal volume of 4% paraformaldehyde and permeabilized with 0.1% Triton X-100 (VWR, Radnor, United States). To block nonspecific binding, PBS containing 10% fetal bovine serum was added to all cells and incubated for 10 min at room temperature. The studied cells (2 × 10^5^) were incubated with 0.25 μg of antibodies and isotype controls for 30–60 min in the dark at room temperature. The following antibodies were used (BioLegend, San Diego, United States): suppressor cells of myeloid origin—APC anti-mouse/human CD11b Antibody, PE anti-mouse Ly-6G Antibody, and FITC anti-mouse Ly-6C Antibody; T regulatory lymphocytes—APC anti-mouse CD4 Antibody, FITC anti-mouse CD25 Antibody, and PE anti-mouse FOXP3 Antibody; CD8+Perf+ T-cytotoxic lymphocytes—FITC anti-mouse CD3 Antibody, PE anti-mouse CD8b Antibody, and APC anti-mouse Perforin Antibody; T helper cells—FITC anti-mouse CD3 Antibody and APC anti-mouse CD4 Antibody; natural killer cells—PE anti-mouse CD335 (NKp46) Antibody, FITC anti-mouse CD3 Antibody, and APC anti-mouse/human CD11b Antibody; dendritic cells—FITC anti-mouse CD80 Antibody and APC anti-mouse CD83 Antibody. The following isotype controls were used (BioLegend, San Diego, United Sates): APC Rat IgG2b, κ Isotype Ctrl, PE Rat IgG2a, κ Isotype Ctrl, FITC Rat IgG2c, κ Isotype Ctrl, FITC Rat IgG2b, κ Isotype Ctrl, PE Rat IgG2b, κ Isotype Ctrl, APC Rat IgG2a, κ Isotype Ctrl, FITC Armenian Hamster IgG Isotype Ctrl Antibody, APC Rat IgG1, and κ Isotype Ctrl Antibody.

Flow cytometry was carried out on a BD FACSAria™ III Cell Sorter.

### Analysis of Activation of Phagocytic Activity of Peritoneal Macrophages in Mice

The phagocytic function of macrophages was assessed using the method presented in [45]. Peritoneal macrophages were isolated from the abdominal cavity of tumor-bearing mice and mice after therapy on days 15 and 22 from the start of the therapy, seeded in a culture plate, and cultured in RPMI-1640 medium (Biolot, Saint Petersburg, Russia) supplemented with 10% fetal bovine serum for 18 h for attachment. Magnetic beads Dynabeads M-280 (Invitrogen, Carlsbad, United States) were added to each well in an amount of 60 μg/well, incubated for 30 min, and washed three times with PBS. Macrophages were photographed in transmitted light using an AxioObserver Z1 inverted microscope (Carl Zeiss Microscopy, Oberkochen, Deutschland); the number of internalized granules (IBN) was counted. The phagocytic activity of macrophages was assessed using the following formula: IBN = number of internalized beads/number of macrophages. Cells were counted manually using the ImageJ program (NIH, Bethesda, United States) in 4 fields with a magnification of ×40; 200 macrophages were counted for each point; the treatments were performed three times.

### MTT Assay

In this study sections, we compared the relative cytotoxicity of the cells for the two samples containing peritoneal macrophages (PMPs) and tumor cells. At the first stage, the changes in the number of both cell populations after co-incubation were determined by counting the number of anchored PMPs and the number of floating tumor cells per several fields of view [5] before and after the incubation. The number of PMPs was found to be unchanged, while the number of tumor cells decreased abruptly. MTT assay was performed for quantitative evaluation of the process. Samples of mouse peritoneal macrophages (0.5 × 10^6^) were incubated with Lewis tumor cells (10^6^) for 24 h. Then, a freshly prepared MTT solution (Aldrich Chemical Company, Milwaukee, United States) was added to the cells to a final concentration of 0.5 mg/ml followed by incubation for 2–3 h in a CO_2_ incubator [46]. After incubation, the cell plate was centrifuged at 400 g for 5 min (Eppendorf, Hamburg, Deutschland) the supernatant was removed, and the resulting crystals were dissolved in 500 μl of dimethyl sulfoxide (VWR, Radnor, United States). Aliquots of 100 μl were taken into a 96-well plate (four replicates for each sample), and the optical density was measured at 550 nm using a Victor X4 plate reader (PerkinElmer, Waltham, United States) by subtracting the background optical density at 616 nm. The survival rate of Lewis tumor cells cultured separately in the medium was considered 100%.

### Statistical Analysis

Statistical analysis was performed using the Statistica 8 software (StatSoft, Tulsa, United States). The validity of differences was evaluated using the Mann–Whitney *U* test or analysis of four-field contingency tables. The revealed differences were considered statistically significant at *p* < 0.05 (Mann–Whitney *U* test) or χ^2^ *P*v < 0.01 (analysis of four-field contingency tables). The graphs show Mean ± SEM or Average ± SD.

## Results

Essential Note. In one of our recent reports, the similar work on determining the same indicative parameters for Lewis carcinoma has been presented [38]. The main topic of that report is quite different from the current one and is devoted to the comparison of several new therapeutic approaches, including the currently reported “Karanahan”. These basic parameters have been determined repeatedly using the congenerous tumor specimens. In the current report, we use the previously obtained results of determining the duration of DNA-repair process with the appropriate reference in the figure legend. All the other indicators are taken from other experiments. Since the tumor is a transplantable stable tumor cell line, all the obtained results are absolutely congruent independently on the time and the number of experiments.

Also, the section devoted to determining the efficacy of the approach under development is provided both with the results of experiments already described in the same report (with the appropriate reference in the figure legend), and, additionally, with those previously unpublished. Such a material presentation way was essential for the generalized (by the totality of experiments) assessment of the seasonal and inter-annual efficiency of the technology under development, discussed in the corresponding section.

### Determination of the Basic Parameters of the Karanahan Approach

#### Internalization of TAMRA-Labeled DNA Probe by Lewis Carcinoma Cells

The main target of the new antitumor approach are CSCs capable of internalizing dsDNA. For this reason, these cells were detected in a solid Lewis carcinoma graft based on internalization of an extracellular TAMRA-labeled DNA probe.

Figure 2A shows a cytological analysis demonstrating the presence of TAMRA+ cells in a Lewis carcinoma tumor. The percentage of TAMRA+ cells (CSCs) in Lewis carcinoma tumors was 12.9 ± 3.1%.

#### Analysis of Time Parameters of Repair Cycle of Lewis Carcinoma Cells Using the Comet Assay After Mitomycin C Injection

To analyze the duration of repair cycle after treating Lewis carcinoma cells with the crosslinking agent mitomycin C *in vitro*, changes in the number of DSBs were assessed in cells using the comet assay. The first phase of interstrand crosslink repair, the NER phase, is characterized by DSB occurrence and accumulation. During the second phase, which is HR, DSBs are repaired. Their disappearance indicates completion of the repair cycle.

The duration of repair cycle in Lewis carcinoma cells after treatment with mitomycin C was found to be 30 h (Figure 2B). The maximum number of DSBs (transition from NER to HR) is observed 18 h after exposure to mitomycin C. This time point corresponds to DNAmix administration in this therapy.

Based on the analysis results, a diagram of triple treatment with a crosslinking agent for Lewis carcinoma was created (Figure 2C). CP was administered with a 2-h shift from the start of the therapy (28 h), which prevented the cells that were subjected to repair first from escaping the arrest.

#### Determination of the Time of Synchronization of Lewis Carcinoma Cells

The time of final treatment in the technology under development leading to complete eradication of CSCs is determined experimentally after three treatments with a crosslinking agent (mitomycin C or CP). Two approaches were used to establish the time schedule: 1) daily analysis of the number of TAMRA+ cells; 2) daily analysis of the cell cycle.

##### Analysis of the Content of TAMRA+ Cells After Triple Exposure to Mitomycin C

Analysis of changes in the content of TAMRA+ cells among tumor cells showed that percentage of TAMRA+ cells decreases on day 4 and then increases on day 5, after which it reaches almost zero level again (Figure 2D).

##### Analysis of Proliferative Activity of Tumor Cells After Triple Treatment of Mice With CP

It was necessary to determine the time of terminal eradication treatment, when the majority or, even, all cells are in G2/M, which is followed by the day when the cells synchronously enter the G1 phase. As we have shown in our previous studies, cell accumulation in G1 coincides with the time of an increase in TAMRA+ cells. This is due to the fact that, at this time point, committed tumor cells undergo apoptosis. TAMRA+ CSCs that have escaped three treatments with the crosslinking cytostatic agent are present in the same amount in the population, which naturally leads to an increase in the percentage of TAMRA+ cells. This cell behavior indicates that the time of terminal eradication treatment, when CSCs are synchronized, has been determined.

CSCs in G2/M do not internalize the dsDNA probe [35]. The reason for this phenomenon is, apparently, a change in general cellular processes associated with cytoskeleton reconstruction due to preparation for/execution of mitosis. The cytostatic agent is delivered to the cell at any cell cycle phase, including G2/M, while the resulting interstrand crosslinks can be determined only in the upcoming G1 and then S phases.

When determining the time regimen for the approach to cancer treatment, the last injection of the cytostatic agent is administered when the cells are in G2/M. DNAmix is administered at a certain time point of the repair cycle, when the number of DSBs reaches its maximum.

To analyze the time point of synchronization of Lewis carcinoma cells, mice were injected with CP at a dose of 100 mg/kg body weight three times with a 28-h interval according to the established regimen (Figure 2C). Analysis of the cell cycle of Lewis carcinoma cells showed that accumulation of cells in G2/M is observed on day 4 (Figure 2E). On day 5, final apoptosis of cells previously synchronized in G2/M and transition of CSCs and remaining committed cells to G1 are observed.

The results obtained from two independent analyzes revealed the time of final treatment with the cytostatic agent: day 4 or 96 h after the first injection. This is the time point at which the minimum content of TAMRA+ cells and synchronous accumulation of cells in G2/M are noted.

Administration of the DNAmix is performed at the moment of transition from NER to HR. For Lewis carcinoma, it corresponded to 18 h from each injection of the cytostatic agent. Thus, the last injection of the DNAmix is conducted on day 5 from the start of the therapy and leads to final eradication of CSCs.

The conducted analysis established the regimen of treatment with CP and DNAmix for Lewis carcinoma (Figure 2F).

### Efficacy of Treating Lewis Carcinoma

**FIGURE 3:**
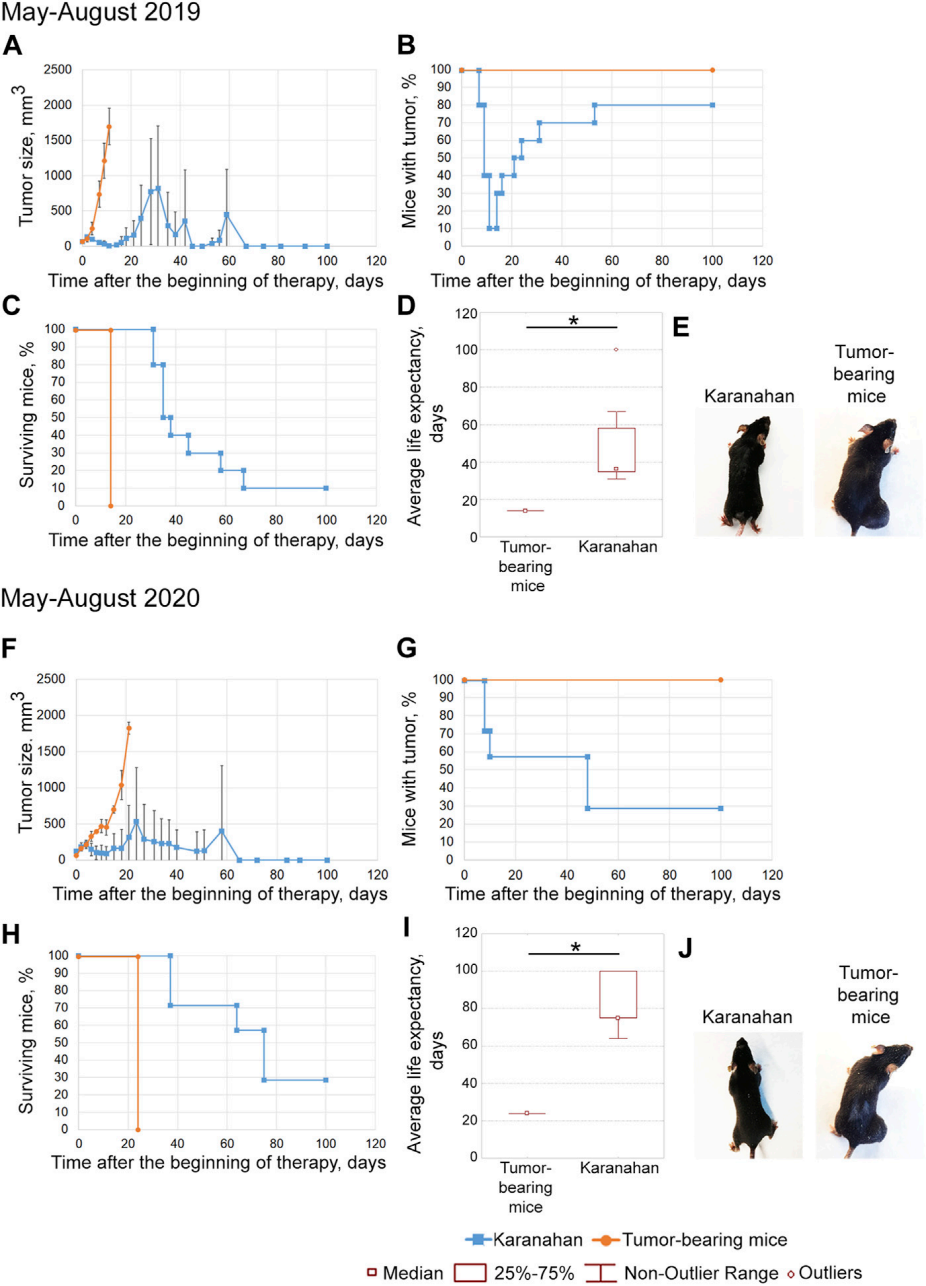
Efficacy of the Karanahan approach in a model of Lewis carcinoma with an intramuscular graft in one femoral region of mice in May–August 2019 **(A–E)** and May–August 2020 **(F–J)**. When a tumor reached the size of 64–125 mm^3^, mice were treated with 100 mg/kg of cyclophosphamide intraperitoneally and 0.5 mg/mouse of the DNAmix intratumorally according to the selected therapeutic regimen. **(A,F)** Comparison of changes in tumor growth in the experimental and tumor-bearing mice groups, Mean ± SD values are shown. **(B,G)** Changes in the number of tumor-bearing mice. **(C,H)** Mouse survival. **(D,I)** Life expectancy in the experimental and tumor-bearing mice groups. *—significant differences from control (tumor-bearing mice), *p* < 0.05; Mann–Whitney U test. **(E,J)** Appearance of experimental animals [**(E)** 11 days after the beginning of therapy; **(J)** 21 days after the beginning of therapy].

**FIGURE 4:**
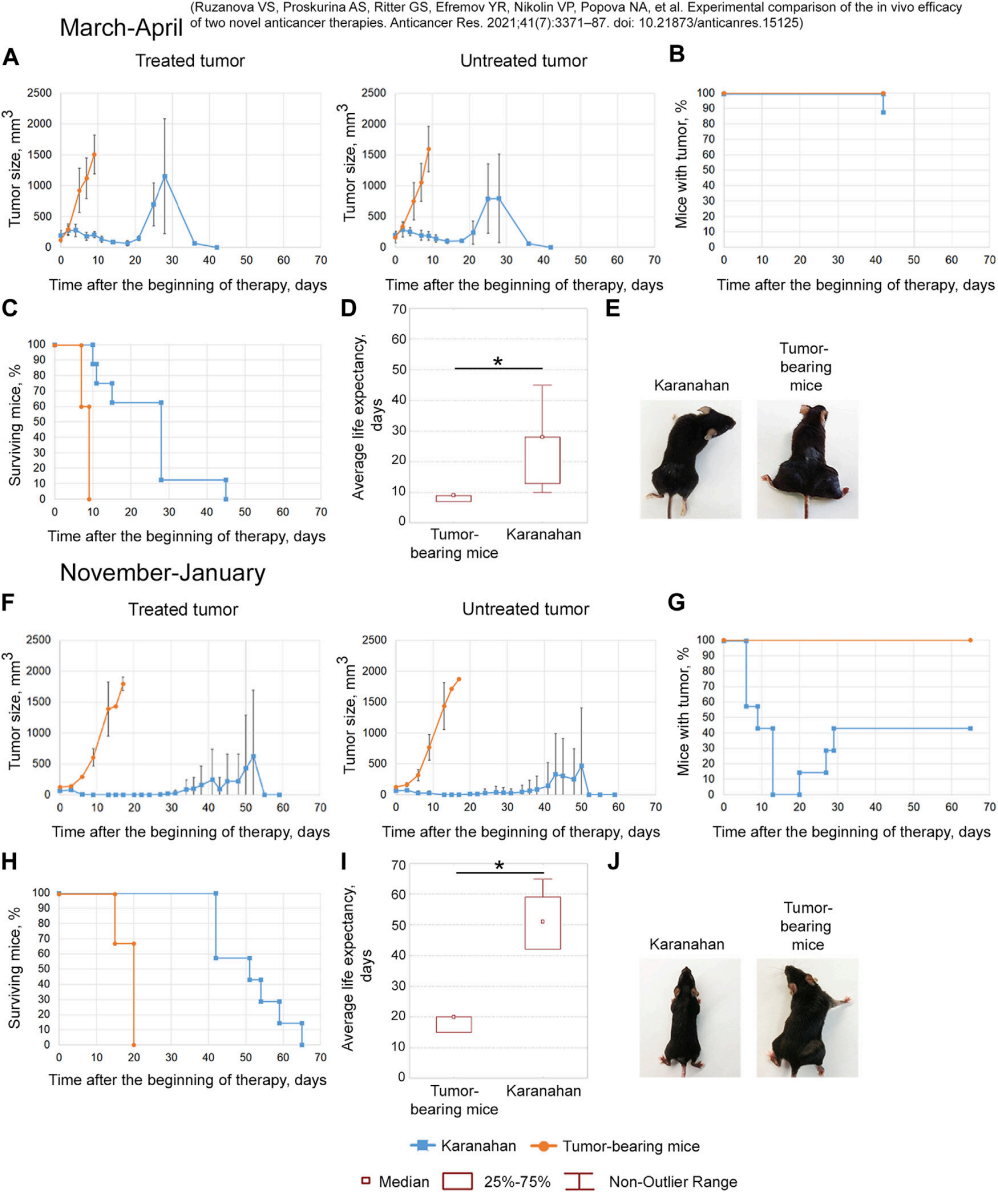
Efficacy of the Karanahan approach in a model of Lewis carcinoma in mice with intramuscular grafts in both femoral regions in March 2019 **(A–E)** [38] and November 2019 **(F–J)**. When a tumor reached the size of either 125–343 mm^3^ (March 2019) or 64 mm^3^ (November 2019), mice were treated with 100 mg/kg of cyclophosphamide intraperitoneally and 0.5 mg/mouse of the DNAmix intratumorally according to the selected therapeutic regimen. DNAmix was injected in only one graft (designated as a treated tumor). **(A,F)** Comparison of changes in tumor growth in the experimental and tumor-bearing mice groups, Mean ± SD values are presented. **(B,G)** Changes in the number of tumor-bearing mice. **(C,H)** Mouse survival. **(D,I)** Life expectancy in the experimental and tumor-bearing mice groups. *—significant differences from control (tumor-bearing mice), *p* < 0.05; Mann–Whitney *U* test. **(E,J)** Appearance of experimental animals [**(E)** 9 days after the beginning of therapy; **(J)** 13 days after the beginning of therapy].

The developed cancer therapy approach was used in several experiments performed in different seasonal cycles and two consecutive years in two modes, with grafting in either one or two femoral regions. When the tumor reached a volume of 64–125 mm^3^, mice were treated according to the selected therapeutic regimen (Figure 2F). The experiments were conducted in May–August 2019 and 2020 (a single graft in the right rear limb, the treatment was applied to the same limb, Figure 3) and in March and November 2019 (two grafts in both rear limbs, the treatment was applied to the right limb only, Figure 4). When the experiment was completed, in March 2019, lung metastases were counted in mice (Supplementary Figure S2). The average life expectancy analysis included fully cured animals that died of natural causes. In order to determine the mean survival in the group, life expectancy of these mice was considered 100 days. The changes in tumor growth in individual mice are presented in Supplementary Figures S1, S2.

#### One Tumor Site

##### May–August 2019

The tumors completely disappeared in 90% of animals by day 11 of therapy. Relapses developed sequentially and were found in 80% of animals by day 53 (Figures 3A,B,E). By day 100 after the start of the therapy, 10% of mice were alive (Figure 3C). The average life expectancy was 14 and 37 days in the control (tumor-bearing mice) and experimental groups, respectively (Figure 3D).

##### May–August 2020

Tumor size was decreased after this therapy; complete tumor eradication has been observed in some mice since day 8. Tumor growth was noted on day 15 in mice that did not exhibit complete tumor eradication (Figures 3F,G). No signs of tumor growth were recorded in 71% of mice in this experiment (Figures 3G,J). A total of 43% of animals died due to the systemic inflammatory response syndrome. By day 100 of the experiment, 29% of the mice were alive; all living animals showed no signs of tumor growth (Figure 3H). The average life expectancy was 23 and 75 days in control (tumor-bearing mice) and experimental mice, respectively (Figure 3I).

#### Two Tumor Sites

To assess the possible effect of a treated tumor site on an untreated one, Lewis carcinoma cells were grafted intramuscularly in two hind legs of mice.

##### March 2019

The antitumor approach resulted in decreased tumor size: tumor in the treatment site was completely resorbed in 13% of animals. In 4 out of 8 mice, tumor recurrence was observed starting from day 21 (Figures 4A,B,E). A total of 38% of mice died due to the systemic inflammatory response syndrome before day 18 (Figure 4C). Dead mice did not show any signs of lung metastases. The revealed number of metastases in the remaining mice of the experimental group was 11 times less than that of the tumor-bearing mice group (Supplementary Figure S2). The average life expectancy was 9 and 28 days in the control (tumor-bearing mice) and experimental groups, respectively (Figure 4D) [38].

##### November 2019

After the therapy, complete tumor resorption was observed 13–20 days after the start of the therapy (Figures 4F,G). Tumors were completely resorbed in both femoral regions in 57% of animals (Figures 4G,J). Spontaneous tumor recurrence was observed in the remaining mice starting from day 20–29, regardless of tumor treatment. The average life expectancy of animals was 20 and 51 days in control (tumor-bearing mice) and experimental animals, respectively (Figures 4H,I).

The efficacy of the technology under development was demonstrated in both experiments. However, complete long-term remission in 60% of experimental mice was achieved only in the experiment conducted in November 2019.

Thus, in four independent experiments conducted consecutively within 2 years, the technology under consideration has been demonstrated to be highly effective in curing “incurable” fulminant experimental murine Lewis carcinoma. In some experiments, a long-term remission (indistinguishable from the complete curing) has been reached in 70% of animals with a single tumor and in 60% with two tumors.

### Effect of Seasonal and Annual Rhythms on the Therapy Efficacy

**FIGURE 5:**
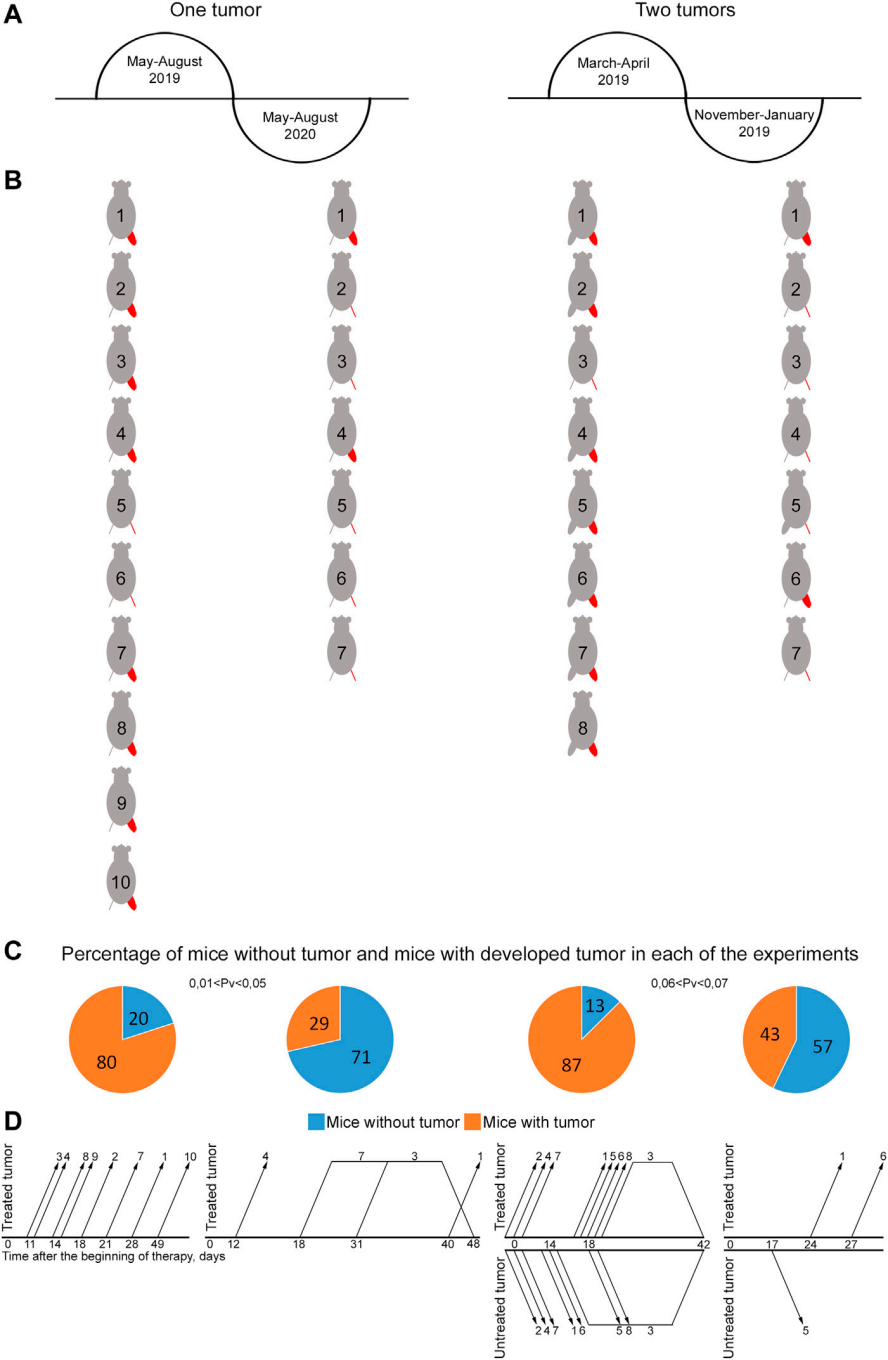
Influence of seasonal and annual rhythms on efficacy of treating mice grafted with Lewis carcinoma. **(A)** Schematic representation of the time of experiments. **(B)** Representation of mice showing the presence or absence of a graft by the end of the experiment. The treated tumor is highlighted in red. A femoral region depicted as a line indicates no tumor. A bulky femoral region represents the presence of tumor. **(C)** Graph showing the percentage of mice with and without tumors. The reliability of the feature difference was assessed using the method of four-fold tables, chi-squared test. **(D)** Schematic representation of occurrence/disappearance of relapses in different femoral regions in experimental mice. The number on the graph corresponds to mouse No. in **(B)**. Changes in the growth parameters of treated and untreated tumors in individual mice in the experimental and tumor-bearing mice groups and a comparison of the size of treated and untreated tumors in individual mice at the terminal experimental point in each group are presented in Supplementary Figures S1, S2.

Initially, there was no intentions to trace the seasonal and inter-annual efficiency of the therapy. In this regard, the size of experimental groups was determined exclusively by the purposes of assessing the therapeutic efficacy, while the exact time frames of experiments were neglected. Nevertheless, the retrospective analysis of the results of four similar experiments, conducted in different seasons of two consecutive calendar years, has revealed the dependence of the effectiveness of therapy both on the season and on the year (Figures 5A,B).

Experiments with two grafts that were carried out in the spring and autumn of the same year demonstrated pronounced changes in tumor reduction efficiency, incidence and development of stable relapses (Figures 5C,D). Tumor efficacy calculated as the number of animals without a tumor was 57% by day 65 and 13% by day 42 in the autumn and spring periods, respectively (0.06 < Pv < 0.07). An analysis of the incidence and stability of relapse development revealed that relapses occurred in three mice and had a stable tendency to development in November 2019. In March 2019, relapses occurred in four animals; they had a stable tendency of development and resulted in animal death from a neoplasm. Furthermore, three mice with a small-sized tumor died, presumably due to the systemic inflammatory response syndrome after therapy. No apparent relationship between relapse occurrence and treatment site was noted. Relapses occurred both in treated and untreated femoral regions after complete tumor resorption in both.

Experiments with one graft that were carried out during two consecutive calendar years in the summer also showed pronounced differences in tumor reduction efficacy and relapse incidence (Figures 5C,D). In 2019, only 20% of mice had no signs of a tumor by day 100 after the beginning of the therapy. Relapses had a stable pronounced tendency to development and led to animal death from a neoplasm. However, in 2020, about 70% of experimental animals had no visible signs of a tumor by day 100 of experiment (0.01 < Pv < 0.05), while only 30% of mice had relapses, which had an unstable tendency to development and further resorbed without tumor re-occurrence. In the other 30% of animals, relapses had a stable pronounced tendency to development; they resulted to animal death from the neoplasm.

Random localization of relapses has been hypothesized to be due to several circumstances.

First, in the case of two tumor sites, they can be assumed to have some interplay at the level of tumor stem cells migration, which occasionally reseed a residual stroma of the resorbed tumors and cause the tumor relapse.

The second hypothesis relates to the immune response, developed due to the treatment applied, both in the case of a single graft and in that of two grafts [4]. Tumor-reactive cytotoxic cells of either innate or adaptive immunity undergo the recruitment to the tumor stroma and inhibit the recurring tumor.

Alternative explanations related to the activity of tissue resident immune cells, e.g., macrophages, which are capable of free migrating into the residual tumor stroma and consequent lysing the recurring tumor, can also be proposed.

Additional experiments on assessing the possibility of migration/dissemination of TAMRA+ (presumably) CSCs of Lewis carcinoma, as well as on assessing the immune response induced by the conducted therapy, have been conducted to clarify the exact mechanism involved.

### Assessment of the Possibility of Migration/Dissemination of TAMRA+ (Presumably) CSCs of Lewis Carcinoma

**FIGURE 6:**
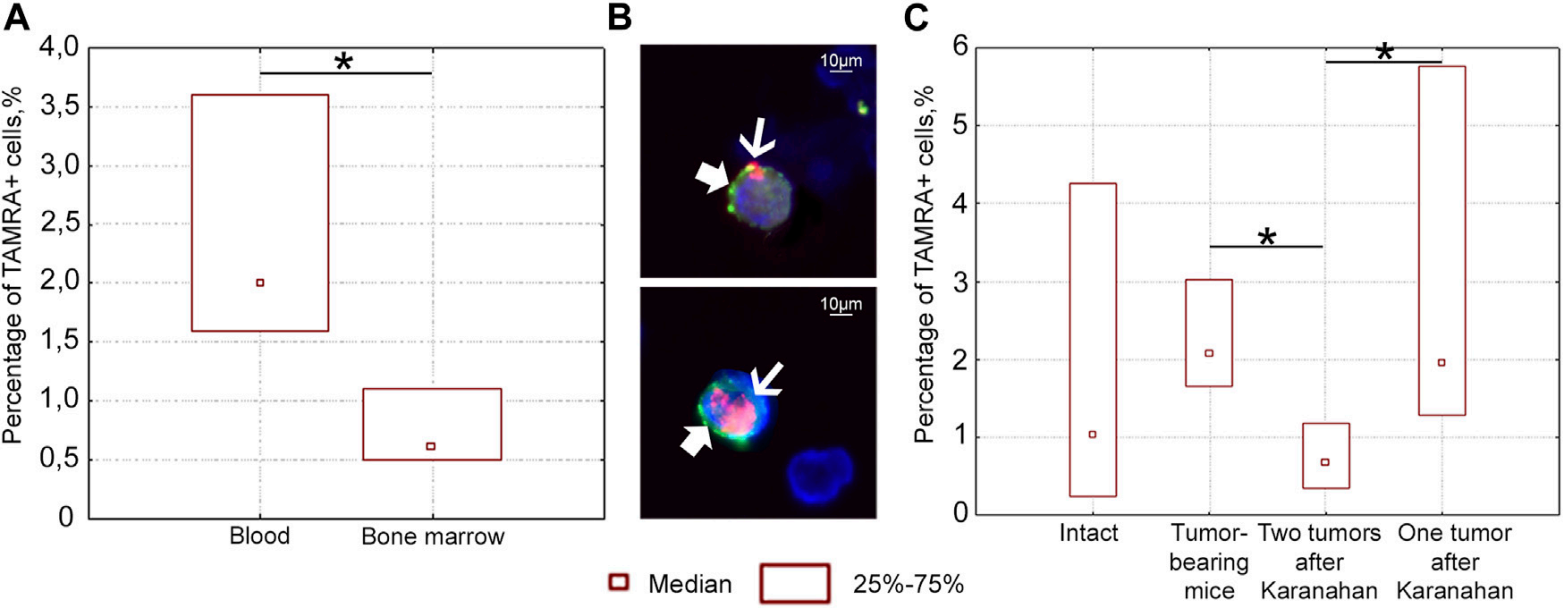
Percentage of TAMRA+ and CD34^+^ cells in the blood and bone marrow of intact and mice bearing Lewis carcinoma, as well as in mice subjected to the Karanahan therapy. **(A)** Percentage of TAMRA+ cells in the blood and bone marrow of intact mice. TAMRA+ analysis in bone marrow and bloodstream of healthy C57BL/6 mice showed that 0.6% of TAMRA+ cells are found in the bone marrow, while about 2.0% of these cells migrate to the peripheral bloodstream in the same mice. **(B)** TAMRA+/CD34^+^ cells in the blood of intact mice when co-stained with anti-CD34 antibodies (bold arrow) and TAMRA-labeled DNA probe (thin arrow). Images were taken at ×120 magnification. Scale bars = 10 µm. This experiment showed that the percentage of TAMRA+/CD34^+^ cells in the peripheral bloodstream of intact mice was 2.4%. Comparison of the results of two independent assessments suggests that practically all TAMRA+ cells of the peripheral blood in C57BL/6 mice also carry the CD34 antigen. The results indicate that TAMRA+/CD34^+^ hematopoietic stem cells and/or their immediate descendants actively migrate across the body [33]. No CD34^+^ cells were found among Lewis carcinoma cells. In other words, in contrast to CD34^+^ blood stem cells of Lewis carcinoma and Krebs-2 CSCs, TAMRA+ Lewis carcinoma cells are positive for only one marker: the ability to capture the TAMRA–DNA probe [15]. **(C)** Percentage of TAMRA+ cells in the blood of intact mice, mice with untreated tumor grafts (Control), and mice subjected to the therapy (with either two or one grafts). *—significant differences between the groups, *p* < 0.05; Mann–Whitney U test.

Three populations of cells positive for TAMRA only, CD34 only, and simultaneously positive for TAMRA/CD34 markers, which mark hematopoietic stem cells and their immediate descendants, were found in the bloodstream of intact mice (Figures 6A,B). This distribution of markers in intact animals did not allow for identifying the origin of bloodstream TAMRA+ cells when analyzing these cell populations in tumor-bearing mice. Thus, there was no reason to compare the migration ability of poorly differentiated cells, bone marrow, and tumor. However, individual analysis of markers revealed the following results.

A series of experiments was carried out to assess the number of TAMRA+ cells after the therapy in the blood of mice with either one or two grafts. The content of TAMRA+ cells was analyzed in the blood on day 6 after the start of the therapy. TAMRA+ blood cell assay assumes that both blood stem cells (as well as their immediate descendants) and Lewis carcinoma stem cells are evaluated together. The percentage of TAMRA+ cells was found to be significantly lower in the blood of mice bearing two tumors, compared both to mice with a tumor in one femoral region and to mice bearing an untreated graft (Figure 6C). This may be due to the fact that two tumor growth sites (TGSs) affect the body in a certain way, and migration/dissemination of stem cells of various origins is inhibited. Moreover, the effect of one tumor is less pronounced, while stem cells are mobilized. A change in the percentage of TAMRA+ cells observed in different treatment regimens clearly assumes potential conditions for both cell release into the peripheral blood and their retention in the bone marrow stroma or tumor.

Taken together, these results suggest the possibility of migration/dissemination of TAMRA+ Lewis tumor cells, which depends on the number of TGSs.

### Assessment of the Immune Response Induced by the Therapy

The results obtained in our studies confirmed that this antitumor approach leads to two main events [1]: induction of massive apoptosis of committed tumor cells [2]; eradication of CSCs from the TGS. These events result in the cure of experimental mice from fulminant malignant experimental tumors [33, 36, 37, 47–49]. CpG oligonucleotides are main factor inducing an immune response in the latest modern approach based on the principle of *in situ* vaccination. CpG oligonucleotides activate tumor-infiltrating dendritic cells. The Karanahan technology also utilizes dsDNA molecules, which, in addition to being involved in repair process in CSCs, also activate the functional properties of antigen-presenting dendritic cells [50–53].

It was suggested that the third event that determines the antitumor effect of the approach to cancer treatment is immune response activation, which, together with apoptosis and eradication of CSCs, creates conditions for curing experimental animals. At the first stage, an exploratory (pilot) study was carried out to determine the time of activation and immunity element that is activated by the therapy (primary immunogram). For more details, see the Materials and Methods section and Supplementary Figure S3. There has been conducted the analysis of the cell populations status with regard to the presence/absence of the residual tumor tissue. This analysis revealed that the residual tumor tissue does not affect the changes in the analyzed populations. The main factor responsible for these changes is the time passed after therapy.

**FIGURE 7:**
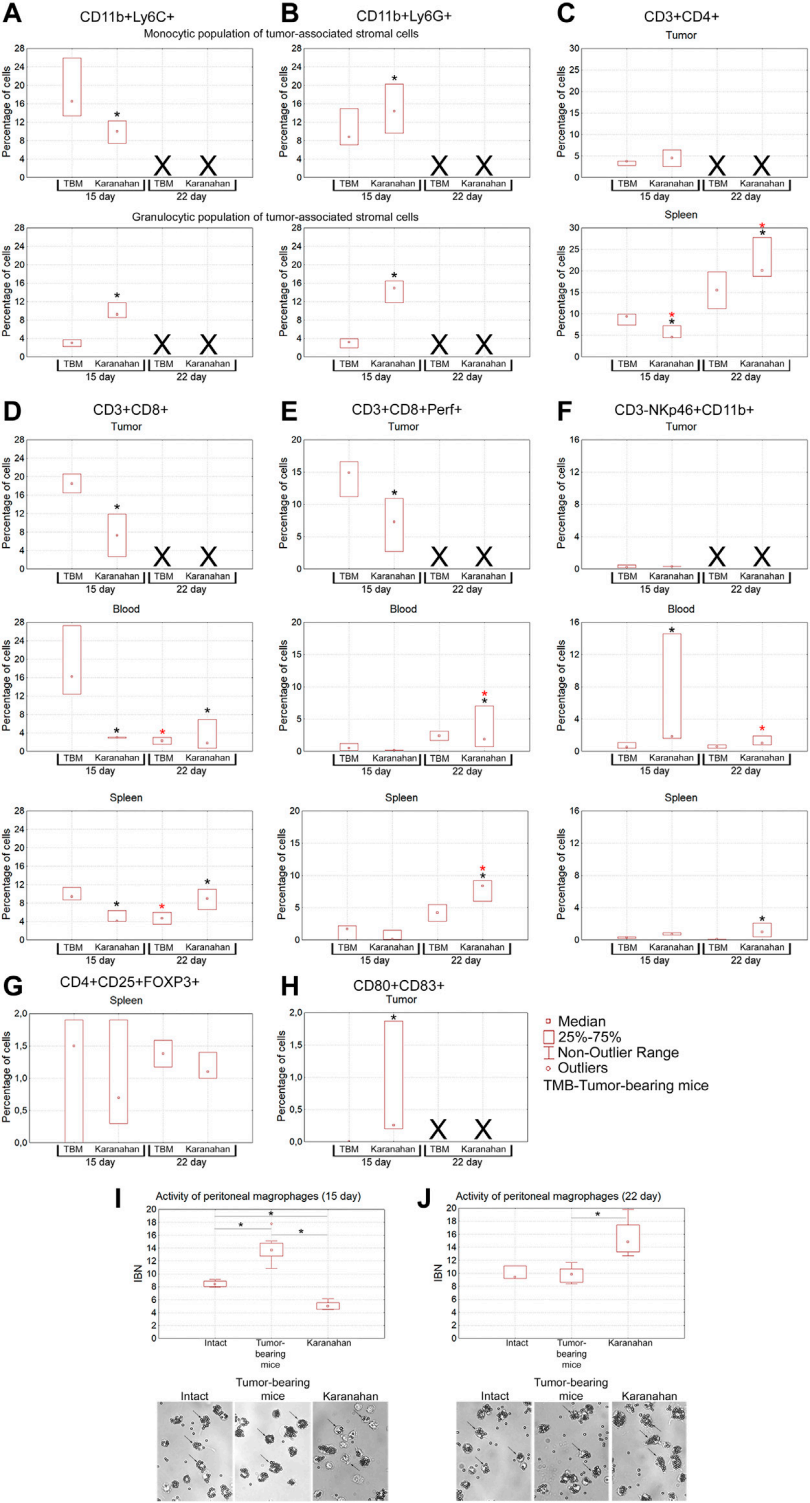
Assessment of the immune response induced in mice with Lewis carcinoma (statistically significant sample of experimental animals) by the Karanahan therapy. Median percentage of populations of myeloid-derived suppressor cells, Thelp, Treg, Tkill, NK, and dendritic cells in the tumor, spleen, and blood isolated from the tumor-bearing and experimental mice on days 15 and 22 after the therapy. **(A)** CD11b+Ly6C+ cells. **(B)** CD11b+Ly6G+ cells. **(C)** CD3^+^CD4^+^ cells. **(D)** CD3^+^CD8^+^ cells. **(E)** CD3^+^CD8^+^Perf+ killer T cells. **(F)** CD3-NKp46+CD11b+ cells. **(G)** CD4^+^CD25+FOXP3+ cells. **(H)** CD80^+^CD83^+^ cells. A black asterisk shows a significant difference between the experimental and tumor-bearing mice groups, a red asterisk indicates a significant difference between the results obtained on a certain day and the result of the previous day of sample collection; confidence level is χ^2^ *P*v < 0.01. Xdenotes that no analysis was performed, since tissue sample was not collected. **(I,J)** Phagocytic activity of peritoneal macrophages measured by their ability to capture magnetic particles. IBN is the magnetic particle capture index. *—significant differences between the groups, *p* < 0.05; Mann–Whitney *U* test. The graphs show medians, interquartile range, and outliers in each group. Images were taken at ×8 magnification.

At the second stage, there has been conducted the similar investigation aimed at the verification of results obtained in the exploratory one (preliminary immunogram) and the estimation of statistical reliability of the determined changes in cell populations. Based on the results of the exploratory experiment, two time points of days 15 and 22 from the experiment beginning have been chosen. For the statistical reliability, the control (tumor-bearing mice) and experimental groups (of 3 mice each) were formed for the each chosen time point. The results obtained are shown in Figure 7. It can be noted that all the patterns detected in the exploratory experiment (Supplementary Figure S3) have been validated for all tested cell populations.

#### Myeloid-Derived Suppressor Cells

Population of suppressor cells of tumor-associated stroma is a mix of myeloid progenitors, which is believed to determine pro-tumor or tumor-reactive status of immune cells infiltrating the tumor site [54, 55]. The population of myeloid progenitors suppresses dendritic cell maturation, natural killer (NK) activity, tumor-reactive or protumor phenotype of tumor-associated macrophages (CD11b+Ly-6C+) and neutrophils (CD11b+Ly-6G+). The following results were obtained (Figures 7A,B). Separate monocytic and granulocytic populations of tumor-associated stromal cells were clearly presented in the tumor. Both populations are positive for macrophage- and neutrophil-specific markers (CD11b+Ly-6C+/Ly-6G+). The presence of CD11b+Ly-6G+ cells in the monocytic fraction can be explained by the immature status of myeloid progenitors of neutrophils lacking lytic granules. The presence of CD11b+Ly-6C+ cells in the granulocytic fraction can be explained by the presence of terminally differentiated macrophages and dendritic cells, which contain the granules and also express the surface CD11b+Ly-6C+ markers.

##### Tumor, Monocytic Population of Tumor-Associated Stromal Cells

On the day 15 from the experiment beginning, the content of CD11b+Ly-6C+ macrophages increased significantly in the group subjected to treatment, indicating the appearance of terminally differentiated macrophages and mature dendritic cells in the tumor node.

##### Tumor, Granulocytic Population of Tumor-Associated Stromal Cells

The content of CD11b+Ly-6G+ fraction significantly increased in the experimental group on the day 15, indicating the recruitment of neutrophils to the tumor node.

It should be noted that tumor-associated macrophages and neutrophils of CD11b+Ly-6C+ and CD11b+Ly-6G+ phenotypes are not always pro-tumor. When determining M2 and N2 phenotypes, the arginase activity of these cells, in addition to specific surface markers, is of paramount importance [56, 57] in preparation.

#### T Helper Cells

Mature T helper cells are the effectors that stimulate and enhance initial immune responses [58, 59]. This cell fraction has the following features (Figure 7C). No significant changes in the cell number in tumors were observed on days 15 and 22 of the experiment. In the spleen, the content of CD3^+^CD4^+^ cells increased significantly on the day 22 from the experiment beginning. These cells are responsible for the activation and maturation of effector cells, and their number increase indicates the onset of the adaptive immune response.

#### CD8^+^ and CD8^+^Perf+ Cytotoxic T Lymphocytes

Injection of a dsDNA in TGS and further stimulation of antigen-presenting properties of dendritic cells of tumor-associated stroma due to tumor-destroying effect of CP is believed to result in *in situ* priming of numerous immature CD8^+^ lymphocytes [60]. This should trigger a T cell response against the vast majority of tumor antigens. Nevertheless, the data obtained indicate that the majority of primed CD3^+^CD8^+^ cells arise not within the tumor, but, most probably, in the “classical” way after migration of activated dendritic cells into lymph nodes. After being primed, immature CD3^+^CD8^+^ cells migrate into the spleen on the day 22, which is the main sign of the establishing adaptive immune response after treatment (Figures 7D,E).

#### NK

NK is the main population of cells able to lyse a tumor without preliminary antigenic recognition [61]. This population of cells is practically absent in the TGS of treated animals. In blood mononuclear cells of experimental mice, the percentage of NK increased significantly. The number of mature NK in splenocytes of treated mice increased significantly on day 22 (Figure 7F).

#### Regulatory T Lymphocytes

CD4^+^CD25+FoxP3+ regulatory T lymphocytes possess a pronounced suppressive activity and normally suppress an immune response after complete neutralization of a pathogen. In the case of a malignant neoplasm, regulatory T lymphocytes are recruited to the TGS to induce anergy of tumor-reactive immune cells, which leads to immunological tolerance and presents one of the ways to avoid immune surveillance by the developed neoplasm [62].

No CD4^+^CD25+FoxP3+ cells were detected in the TGS during the entire study period (Figure 7G). The presence of regulatory T lymphocytes in the spleen indicates the activation of feedback control mechanisms suppressing excessive escalation of activated immune responses in the experimental group.

#### Dendritic Cells

Mature antigen-presenting cells are the inducers of the adaptive immune response, including the anti-tumor one. The content of mature dendritic cells (CD80^+^CD83^+^) in the tumor node was assessed. It turned out that in all three treated mice, a high level of dendritic cells was detected on day 15 after treatment (Figure 7H). At the same time, no these cells were detected in the tumor-bearing mice group.

#### Peritoneal Macrophages

Peritoneal macrophages were analyzed as a population of cells that forms an immune barrier outside the circulatory system. In this part of the study, activity of peritoneal macrophages was assessed using a standard test for their ability to capture magnetic particles (phagocytic activity) [45]. It has been shown that the fraction of macrophages capable of capturing magnetic particles in mice subjected to the therapy significantly increased within the days 15–22. In this experimental group, macrophages underwent morphological changes, became larger, while the IBN phagocytic index increased from 5 (day 15) to 15 (day 22) (*p* < 0.05, Mann–Whitney *U* test) (Figures 7I,J).

Thus, based on the two independent experiments, we can state that therapy in mice carrying a Lewis carcinoma graft activates the antitumor immune response. There is an increase in the number of NK cell populations in the blood, tumor, and spleen, content of killer T cells and T helper cells in the tumor and spleen, and percentage of CD11b+Ly-6C+ and CD11b+Ly-6G+ cells in the tumor. A population of mature dendritic cells is found in the tumor. Identification of dendritic cells and cytotoxic T lymphocytes in the TGS may indicate *in situ* priming of naive CD8^+^ cells and formation of a population of adaptive immune response cells in the tumor [60]. Different percentages of both CD11b+Ly-6C+ macrophages and T helper cells than those of the tumor-bearing mice group indicates involvement of macrophage activation in the immune response.

## Discussion

### Lewis Carcinoma Therapy Using the Karanahan Approach

Indicative parameters of the approach have been determined for Lewis carcinoma in the present study. Using the obtained indicative parameters, we managed to cure 57% of animals bearing tumors in two femoral regions (measurements ended on day 65) and 71% of animals bearing tumors in one femoral region (measurements ended on day 100). Our experience of working with Krebs-2 carcinoma shows that if a relapse has not been formed before day 90 from the start of the experiment, then it will not be formed until the natural death of the animal. That is, a mouse is considered to be cured of a grafted tumor if a secondary tumor does not occur before day 90. Lewis carcinoma is an aggressive fast-growing cancer that is believed to be incurable. The life expectancy of untreated animals bearing this tumor is about 10–30 days. The obtained result indicates the efficacy of the developed approach for treating Lewis carcinoma, which has been previously tested in nine experimental models [33, 36, 37, 39, 47, 49].

### Seasonality of Changes in Efficacy of the Karanahan Therapy in a Model of Lewis Carcinoma

Seasonal changes and changes associated with year alternation in the efficacy of the therapy were found in in the current study.

Seasonal and circadian changes have been known and characterized for the biological systems [63–66]. Seasonal changes in microRNA gene expression [67, 68] and tumor necrosis factor production [69] have been described. Seasonal dependence of the synergistic action of CP and a human native DNA preparation was shown in hematopoietic stem cells in our previous experiments. In winter, the lymphoid hematopoietic origin is deformed due to the synergistic action, and a period of time called “window of death” is formed, which ends in summer [10]. A seasonal correlation in the incidence of primary cancers associated with vitamin D level [70] and seasonal correlation in the incidence of postoperative lung metastases in breast cancer [71] were described. Many biochemical parameters (serum melatonin, steroid receptor level) of breast cancer are seasonal [72,73]. There are also many experimental studies (seasonal cycles of testicular activity, cyclical histogenesis, impact of autumnal environment on encephalic cell proliferation) characterizing seasonal and circadian changes in proliferative activity of various types of cells, including stem cells [74–80].

These alterations are believed to be due to changes in seasonal frequency and amplitude of cortisol release by the hypothalamic-pituitary-adrenal system. The winter rhythm with a high amplitude leads to maximum synchronization and a time shift in cell cycle phases compared to the summer [81–85].

Since the process of repairing any damage to the chromatin is directly related to proliferation, we can assume that, seasonal variability and year-to-year alterations found for Lewis carcinoma are due to a change in proliferative cycle duration, which is the reason for fundamental changes in the main platforms of the antitumor approach under consideration.

### Spontaneous Relapses, Possible Causes

Analysis of the results of the therapy in mice bearing two tumors revealed spontaneous relapses, independent of whether the tumor was treated with a DNA preparation. This phenomenon may be due to two circumstances: 1) communication between TGSs through CSC migration/dissemination; 2) unevenly developed immune responses.

#### Release of Stem Cells Into the Bloodstream

##### Migration/Dissemination of CSCs

Continuous traffic of hematopoietic stem cells between the bone marrow and the blood is assumed one of the mechanisms for redistributing precursors and replenishing depleted bone marrow niches, which contributes to normal hematopoiesis. Migration of hematopoietic precursors maintains the balance of stem cells in the bone marrow and distant body parts. In addition, circulating hematopoietic stem cells patrol tissues and repair them after infections and physical damage [86–88]. Other types of stem cells circulate in the peripheral blood: mesenchymal stem cells, endothelial progenitors, and small embryonic stem cells. Normally, stem cells leave their niches after asymmetric division. The forced induced exit of stem cells from their niches into the blood also occurs during pharmacological mobilization, after which the number of stem cells increases ˃10–fold compared to the normal range. The content of the entire population of stem cells circulating in the bloodstream depends on circadian rhythms, presence of trauma and heavy loads [86, 88–90].

Tumors without clinically evident metastases can also form a pool of circulating (migrating) tumor cells. This is a heterogeneous population that includes a separate minor subpopulation of CSCs amounting to 0.01%. These cells, as well as their stemness and invasiveness, play a crucial role in metastasis. The number of circulating CSCs correlates with tumor aggressiveness [91, 92].

The present study has demonstrated that both types of poorly differentiated cells, hematopoietic stem cells and Lewis carcinoma CSCs, can simultaneously be present in the bloodstream. This means that a certain communication can exist between the TGS through CSC migration. CSCs that have survived the treatment migrate to the tumor-free site, where the capsule and stroma of destroyed tumor cells are preserved, populate stromal niches, and induce a relapse. The reasons for uneven and selective distribution of CSCs between TGSs in this case are unknown and require further research.

In addition, a certain type of relationship can occur between hematopoietic and cancer stem cells [93], which can affect a change in biological properties of both populations of poorly differentiated cells (change in the status of hematopoietic stem cells towards their tumorigenicity, as well as increased invasiveness and aggressiveness of CSCs). This interaction can also explain logical inconsistency in experiments, when an untreated graft grows more slowly than a treated one.

#### Activation of Immunity

The results obtained demonstrate that the therapy activates an antitumor immune response and this activation is the third therapeutic platform (along with CSC eradication and induction of massive apoptosis of committed tumor cells) of the antitumor effect of the new cancer treatment approach. As it follows from the analysis of changes in immune cell populations, both vectors of the immunity are activated in experimental mice: the innate (including macrophages outside the circulatory system) and the adaptive immunity. The observed uneven changes in graft size and non-systemic development of relapses in different femoral regions can be the phenotypic manifestations of such activation.

We assumed that, in case if drug treatments induce an immune response, the following changes in tumor development might be observed:

1. Similar and different from zero parameters of the total tumor volume for both femoral regions within the same group may indicate either the absence of any immune response if the values are close to the control (tumor-bearing mice) ones or insufficient innate and adaptive immune responses if they somehow differ from the control (tumor-bearing mice) values;
2. Close to zero total parameters for both femoral regions indicate a significant innate and adaptive immune responses;
3. If the total parameters in the treated site within the same group are lower than those of the untreated one, it indicates the prevalence of innate immune response in the treatment site and insufficient adaptive immune response at the untreated site, in case if untreated tumor in an experimental mouse is smaller than that of the control (tumor-bearing) mouse;
4. If the total parameters in the treated site in the same group are lower than those of the untreated one, while the untreated tumor in an experimental mouse is similar in volume to untreated tumor of the control (tumor-bearing) mouse, it indicates an innate immune response in the treatment site and the absence of an adaptive immune response at the untreated site;
5. If the total parameters of untreated site are lower than those of treated site within the same group, while tumors on both femoral regions of experimental mice are smaller in volume than those in control (tumor-bearing) mice, it indicates an adaptive immune response in untreated site and an innate immune response at the site of treatment;
6. If the total parameters of untreated site are lower than those of treated site within the same group, while treated tumors of experimental mice are equal in volume to those of control (tumor-bearing) mice, it indicates an adaptive immune response in untreated site and the absence of an innate immune response at the site of treatment.

Thus, induction of immune responses could be assessed by measuring the average size of treated and untreated tumors in the control (tumor-bearing mice) and experimental groups (Table 1).

**TABLE 1:** Average tumor sizes (mm^3^) in treated and untreated sites in control (tumor-bearing) animals and after the Karanahan therapy.

Experiment	Group	Treated femoral region	Untreated femoral region
March 2019	Tumor-bearing mice	1357	1315
	Karanahan	870	918
November 2019	Tumor-bearing mice	2684	2730
	Karanahan	714	392

Tumor volumes were almost equal at two sites in the tumor-bearing mice group in both experiments, which indicates the absence of an immune response. Insufficient activation of both innate and adaptive immune responses was noted in mice in March 2019 after the therapy. In November 2019, the therapy resulted in activation of an immune response, which included both innate immunity in the treatment site and adaptive immunity in untreated site in mice.

Unpredictable manifestation of the innate and adaptive immune responses in the form of unexplained spontaneous change in the tumor size in different femoral regions may be the cause of spontaneous relapses.

The conducted study indicates that both variants can be considered in case of spontaneous relapses: dissemination of CSCs of Lewis carcinoma and divergent induction of immune responses in the grafts.

The current research work raises multiple questions regarding the mechanisms determining the described therapeutic effects of the approach. One of the main issues of how CSCs internalize extracellular dsDNA has been resolved, and currently three reports, describing the factors and mechanisms of this process, are in the process of submission. It is established that negative surface charge of tumor cells, heparin-binding domains, including clusters of positively charged amonoacids, in the structure of glycocalyx glycoproteins/proteoglycans, glycosylphosphatidylinositol-anchored proteins and scavenger receptors are the factors contributing to the internalization of extracellular dsDNA fragments into CSCs. Bound fragments are transported via caveolar/clathrin-dependent mechanism. Other questions, primarily those of the mechanisms of interference of the internalized dsDNA fragments in the DNA repair process, which determine the death of CSCs, require further experimental solutions.

## Data Availability

The original contributions presented in the study are included in the article/Supplementary Material, further inquiries can be directed to the corresponding author.

## Abbreviations

CP, cyclophosphamide; CSC, cancer stem cell; DSB, double-strand break; dsDNA, double-stranded DNA; HR, homologous recombination; IBN, number of internalized beads/number of macrophages; ICL, interstrand crosslink; NER, nucleotide excision repair; NK, natural killer; TAMRA, carboxytetramethylrhodamine; TGS, tumor growth site.
