# Supplementary material for: Chronometric Administration of Cyclophosphamide and a Double-Stranded DNA-Mix at Interstrand Crosslinks Repair Timing, Called “Karanahan” Therapy, Is Highly Efficient in a Weakly Immunogenic Lewis Carcinoma Model
Source: Pathol Oncol Res. 2022 May 27;28:1610180. doi: 10.3389/pore.2022.1610180 (PMC9185167; doi:10.3389/pore.2022.1610180)
Supplement: Supplementary file 1 [file DataSheet1.docx]

**
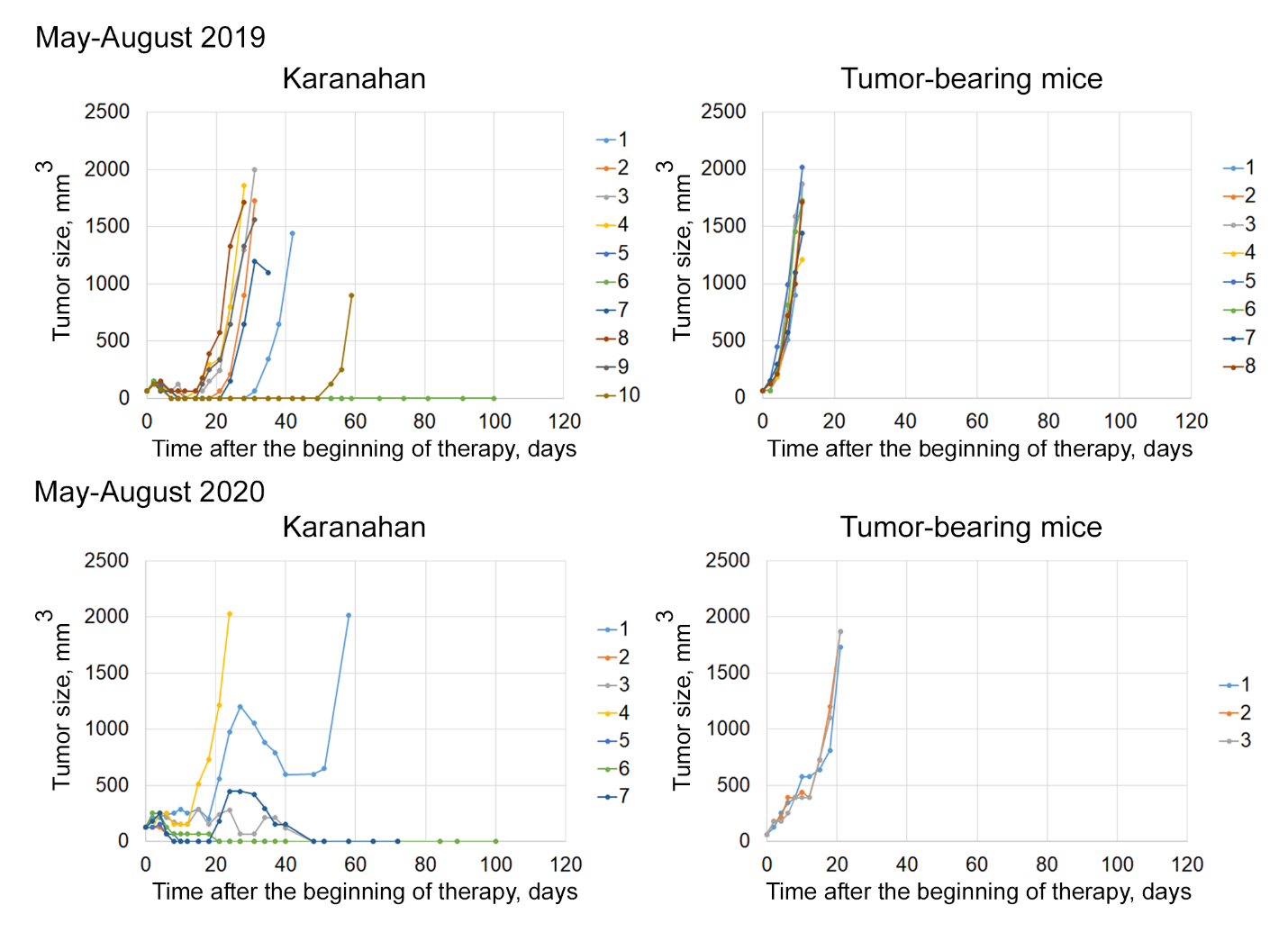
**

**Supplementary Figure S1.** Efficacy of the Karanahan approach in a Lewis carcinoma model in mice with an intramuscular graft in one femoral region in May–August 2019 and May–August 2020. The graphs show individual changes in tumor growth in mice.

**
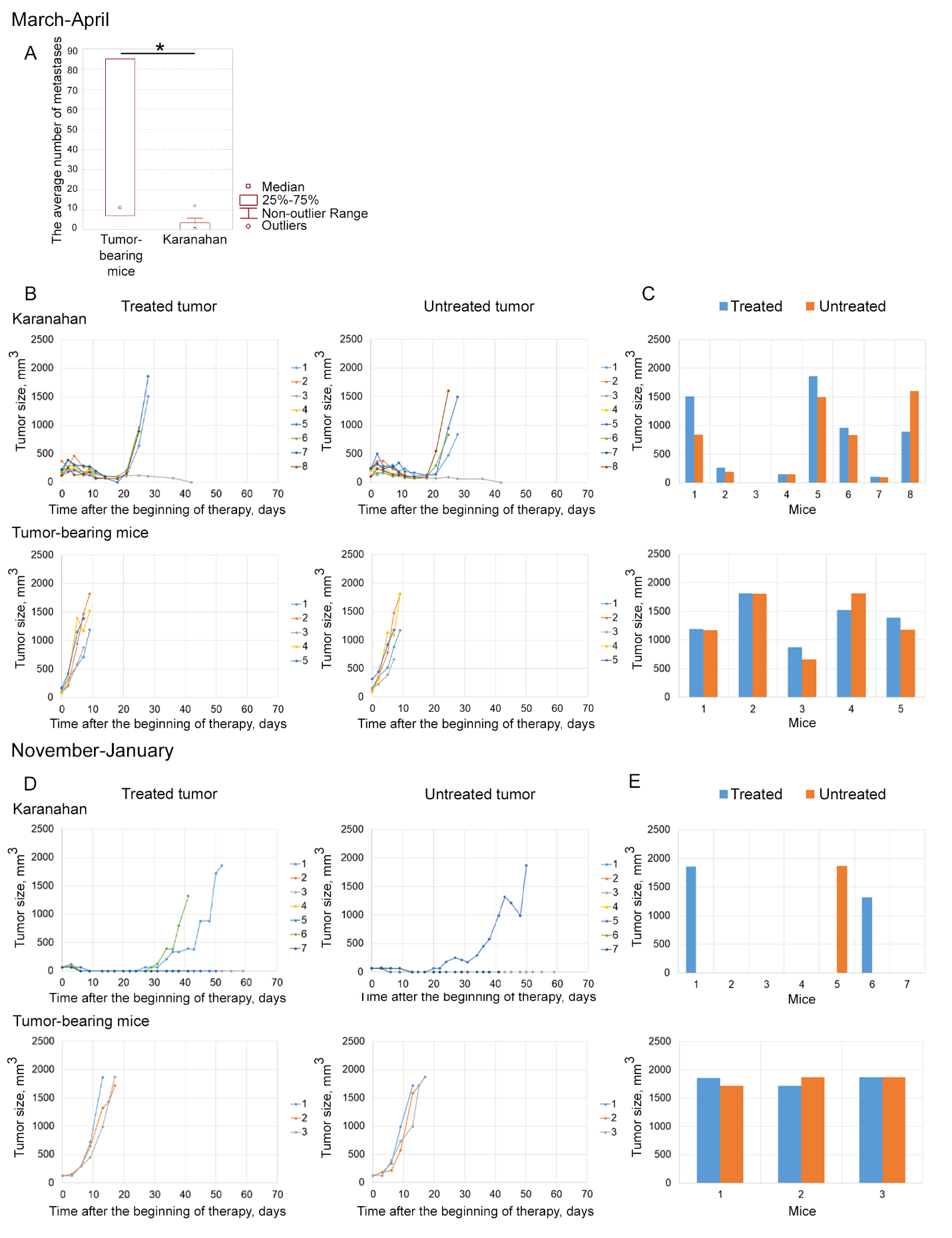
**

**Supplementary Figure S2.** Efficacy of the Karanahan approach in a Lewis carcinoma model in mice with an intramuscular graft in both femoral regions. **(A)** Number of metastases in the control (tumor-bearing mice) and experimental groups. **(B)** and **(D)** Individual changes in growth of treated and untreated tumors in experimental and tumor-bearing mice. **(C)** and **(E)** Comparison of the size of treated and untreated tumors individually in mice of each group at the terminal experimental point.


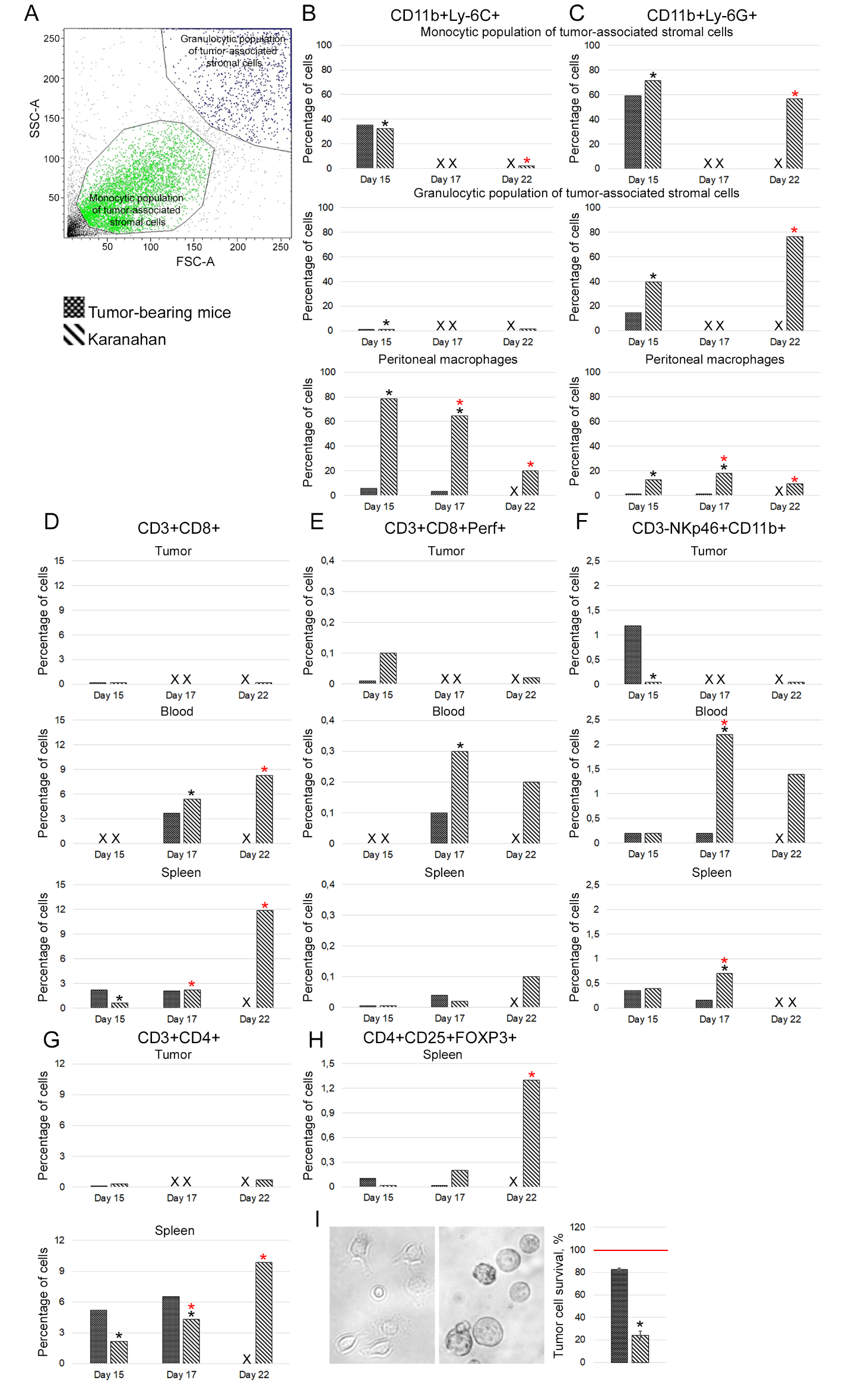


**Supplementary Figure S3.** Activation of various populations of cells involved in the immune response using the new technology "Karanahan". Primary immunogram. **(A)-(C)**. *MDSC.* This population is represented by monocytic and granulocytic populations of tumor-associated stromal cells carrying the CD11b marker. We compared subpopulations of monocyte and granulocyte fractions and determined the number and ratio of CD11b+LycC+ macrophages and CD11b+LycG+ granulocytic neutrophils in the tumor of control (tumor-bearing mice) and experimental animals. We also evaluated these markers in the general population of peritoneal macrophages. Separate monocytic and granulocytic populations of tumor-associated stromal cells were clearly presented in the tumor. *Tumor, monocytic population of tumor-associated stromal cells.* On day 15 of experiment, the number of CD11b+LycC+ macrophages was the same in both groups and amounted to ~ 35%. The number of cells with this phenotype dropped sharply to its minimal percentage of 2% in the experimental group on day 22. The number of CD11b+LycG+ neutrophils also decreased, although less significantly, from 71 to 57% in the experimental group. *Tumor, granulocytic population of tumor-associated stromal cells.* The number of both populations in the experimental group increased with time: from 0.6 to 1.5% and from 40 to almost 80% for CD11b+LycC+ and CD11b+LycG+, respectively. A sharply increased neutrophil percentage indicates a pronounced migration of cells to the TGS. The number of CD11b+LycG+ neutrophils was almost three times higher than that of the population of neutrophils in the tumor-bearing mice group. *Peritoneal macrophages*. Peritoneal macrophages were analyzed as a population of cells that forms an immune barrier outside the circulatory system. The number of peritoneal CD11b+LycC+ macrophages in treated mice was 80% on day 15, it gradually decreased to 65% on day 17 and reached 20% on day 22. These cells constituted up to 3–6% in the tumor-bearing mice. The percentage of CD11b+LycG+ neutrophils in experimental mice practically did not change and constituted 10–20% of the total population of peritoneal cells; these cells accounted for 1.3% in the tumor-bearing mice. **(D)-(E)**. *CD8+ and CD8+Perf+ cytotoxic T lymphocytes*. We assessed tumor, spleen, and blood mononuclear cells. The percentage of CD3+CD8+Perf+ cells in the tumor was ~ 0.1 vs 0.01% in tumor-bearing mice on day 15. Cell content was 0.3 vs 0.1% in tumor-bearing mice in the blood and 0.02 vs 0.04% in tumor-bearing mice in the spleen on day 17. On day 22, the percentage of cytotoxic T lymphocytes in the tumor decreased to 0.02%; the content of CD3+CD8+ Perf+ cells decreased to 0.2% in the mononuclear cell fraction and increased to 0.1% in the spleen. These changes indicate that cytotoxic T lymphocytes are formed and actively spread on day 17 and grow in number fivefold in the spleen by day 22. The same percentage (0.2%) of immature CD3+CD8+ cells was found in the tumor of both tumor-bearing mice and experimental animals on day 15, which did not change for 7 days. The number of cells carrying the same markers in the blood reached 5.4 vs 3.7% in the tumor-bearing mice on day 17 and increased to 8.3% on day 22. The following pattern of cell distribution was observed in the spleen: 0.6 vs 2.2% in the tumor-bearing mice on day 15, 2.2 vs 2.1% in the tumor-bearing mice on day 17, and 12% on day 22. We assume that the two events occur at the same time. Firstly, a small number of CD3+CD8+ cells are formed in the tumor during in situ treatment and spread through the bloodstream to reach the spleen and other body structures. Secondly, based on the data obtained, the majority of primed CD3+CD8+ cells originate not from the tumor but are rather formed via the conventional pathway, i.e. after migration of activated dendritic cells to the lymph nodes. After priming, immature CD3+CD8+ cells migrate to the spleen by day 22 of the experiment, where their number increases 20-fold (12%). This fact is the main sign of an adaptive immune response induced by treatment. **(F)**. *NK.* This population of cells is practically absent in the TGS of treated animals, while the content of mature NK in the tumor of tumor-bearing mice reaches 1.2%. In blood mononuclear cells of experimental mice, the percentage of NK increases from 0.2% on day 15 to 2.2% on day 17 and slightly decreases to 1.4% on day 22. The number of mature NK in splenocytes of treated mice increases from 0.4 to 0.7% on days 15 and 17, respectively. **(G)**. *T helper cells.* No significant changes in the cell number were observed on days 15 and 22 of the experiment. The percentage of CD3+CD4+ cells was 0.3 and 0.7% in treated mice on days 15 and 22, respectively, and 0.1% in the tumor-bearing mice on day 15, which may indicate their proliferation in tumors of experimental animals. A consistent increase in the number of CD3+CD4+ cells from ~ 2% on day 15 and ~ 4% on day 17 to 10% is observed in the spleen on day 22, while their content in the tumor-bearing mice group remains at 5–6%. This type of cells is responsible for secondary activation and maturation of effector cells, and an increase in their population in the spleen may indicate the onset of an adaptive immune response. **(H)**. *Regulatory T lymphocytes.* No CD4+CD25+FoxP3+ cells were detected in the TGS during the entire study period. These cells are found in the spleen of experimental mice at 0.2% on day 17 and grow in number to 1.3% by day 22. The presence of regulatory T lymphocytes in the spleen indicates activation of feedback control mechanisms suppressing excessive escalation of activated immune responses in the experimental group. A black asterisk indicates a significant difference between the experimental and the tumor-bearing mice groups, a red asterisk shows a significant difference between the results obtained on a certain day and the result of the previous day of sample collection; confidence level is χ2 *P*v < 0.01. Х denotes that no analysis was performed, since tissue sample was not collected. **(I)**. *Evaluation of cytolytic activity of blood mononuclear cells, splenocytes, and peritoneal macrophages against Lewis carcinoma cells by the MTT assay.* The main indicator of developed antitumor activity in a particular population of immune cells is their direct cytolytic effect on tumor cells. We performed a series of experiments to determine the efficacy of the cytotoxic effect of blood mononuclear cells, splenocytes, and peritoneal macrophages on Lewis carcinoma cells. In this study sections, we compared the relative cytotoxicity of the cells for the two samples containing peritoneal macrophages (PMPs) and tumor cells. Blood mononuclear cells and splenocytes were shown to lack a pronounced cytolytic activity against tumor target cells (data not shown). On day 15, the studied populations of cells and target Lewis carcinoma cells were mixed in a 1/2 ratio and incubated for 24 hours. As it followed from the cytological analysis (attached macrophages and free-floating Lewis carcinoma cells), a significant cytoreducing effect of the non-attached cells population (Lewis carcinoma tumor cells) was observed. Peritoneal macrophages had a pronounced antitumor effect in a group of treated mice. MTT assay results demonstrated that about 80% of tumor cells were lysed six days after complete therapy. No antitumor activity of peritoneal macrophages was detected on day 31 after complete therapy. The survival rate of Lewis carcinoma cells (in the absence of effector cells) is considered 100% and indicated by a red line. A black asterisk indicates a difference from control (tumor-bearing mice). The confidence level is *p* < 0.05 for all comparisons; Mann–Whitney *U* test. The figure also shows cytological photographs of peritoneal macrophages and tumor cells. Images were taken at x24 magnification.
